# A Case and Intervention for Older Adult Sexual Mistreatment

**DOI:** 10.1177/00912174241276597

**Published:** 2024-08-30

**Authors:** Renee J. Flores, Cristina Murdock, John Halphen, Carlos Reyes-Ortiz, Jason Burnett

**Affiliations:** 1 Department of Internal Medicine, 12339 University of Texas Health Science Center at John P. and Katherine G. McGovern Medical School, Houston, TX, USA; 2 Florida Agricultural and Mechanical University, Tallahassee, FL, USA

**Keywords:** sexual mistreatment, older adult, sexual abuse

## Abstract

**Background:**

The sexual mistreatment of older adults is a significant public health issue; like other forms of sexual violence, it is more prevalent than officially reported. Survivors often hesitate to speak out due to feelings of embarrassment, fear, or potential cognitive impairments. Moreover, the concealment of such mistreatment, coupled with societal stigmas surrounding aging and sexuality, creates challenges in recognizing sexual mistreatment during routine or emergency room visits.

**Purpose:**

This paper provides a framework for informing physicians, psychiatrists, and other healthcare providers on best practices screening, detection, and management of sexual mistreatment in older adults.

**Methods:**

A literature review of older adult sexual mistreatment articles between 2005-2024 was conducted.

**Results:**

Medical professionals and psychiatrists are well-positioned to raise awareness and identify sexual mistreatment in older adults and manage such situations when suspected.

**Conclusion:**

Assessing suspected sexual mistreatment can improve detection, responsiveness, and patient safety for older adults through an interprofessional approach.

Like childhood sexual abuse, older adult sexual mistreatment is a global and national public health concern. Older adult sexual mistreatment is underreported and imposes a range of potentially harmful physical and psychological consequences. Underreporting and identification stem from survivors’ shame and embarrassment in sharing and health professionals’ lack of awareness, assessment, and interviewing skills regarding older adult sexual mistreatment. This article provides a comprehensive overview of older adult sexual mistreatment, offering primary care physicians and psychiatric clinicians strategies for identifying salient and subtle signs of older adult sexual mistreatment and trauma-informed approaches to engage with and respond to older adult sexual mistreatment survivors.

## The case of Mary

Mary is a 60 year-old woman with epilepsy and Intellectual Development Disorder (IDD), neurocognitive diseases of dementia, depression, and psychosis, who presents to the emergency room after a reported fall from her wheelchair. She lives in a personal care home with three other individuals. She has her own room, needs assistance with transfers, and primarily uses a wheelchair. One of the housemates is violent, with a history of mental illness, and is known to bring her boyfriends into the personal care home. In the emergency department, she had ecchymosis around both eyes, fractured femur and neck, with spinal cord damage. The caretaker at the personal care home did not report any problems and could not account for the ecchymosis. Since injuries were not consistent with a fall reported by the facility, the patient’s guardian asked for a genital examination. One-centimeter tears were located at 3 o’clock and 7 o’clock, with blood noted at the vaginal opening. The patient later died due to complications of her injuries.

How would you evaluate and treat a patient with a similar scenario?

## The clinical problem

Mistreatment of older adults significantly impacts approximately 10% of those 60 years old, with 0.8% for sexual mistreatment.^1,2^ This number is likely an underestimation, considering the suspected exponentially high number of unreported cases of rape, sexual assault, and intimate partner violence occurring yearly. Nationally reported cases exceed 5 million older individuals,^3^ highlighting the urgency to increase awareness about the prevalence of older adult sexual mistreatment, whether it is in the presence or absence of physical injury.

State statutes define older adult mistreatment individually, encompassing physical, emotional, financial, caregiver, or sexual mistreatment.^4^ Specifically, sexual mistreatment is nonconsensual sexual contact or unwanted sex talk of any kind; this also includes coercing an older adult to witness sexual behaviors. The term “sexual mistreatment” is favored over “sexual abuse,” as it covers a broader range of mistreatments, as well as deliberate actions.^5,6^

The U.S. Preventive Task Force (USPSTF) recommends screening for intimate partner violence (IPV), acknowledging the underreporting of the older adult population data, with as little as 1 in 5 or fewer cases reported.^7^ Although sexual mistreatment is seemingly the least reported, literature reviews indicate that it is not the least common. The socio-ecological model provides reasoning for why sexual mistreatment is perhaps common but less reported. At the intra-personal level, survivors may feel ashamed or embarrassed, leading them to conceal incidents. At the interpersonal level, a common misconception is that rape and sexual violence are about attraction. Still, studies have debunked this myth, revealing that intentions reflect power and control urges. Although sexual-related crimes are commonly more violent than other crimes, older adult sexual mistreatment more often occurs in the older adult’s residence by someone the older adult knows, lowering the likelihood of detection by authorities, and increasing the possibility of shame, embarrassment and concealment by the older adult. This may feed the community and societal biases that sexual mistreatment is uncommon because ageism depicts older adults as less attractive and desired targets for sexual mistreatment. These factors undoubtedly contribute to the lack of health professional training and practices regarding screening and responding to older adult sexual mistreatment.

Indicators of older adult sexual mistreatment can be subtle, presenting as withdrawn or avoidant behavior. Still, they can also be pronounced with apparent injuries and even death. Behavioral changes have been linked to substance use,^5^ often as a coping strategy, and depression, a psychological consequence of the mistreatment.^8^

In long-term care facilities, like nursing homes and assisted living facilities, more than 16 000 complaints of sexual mistreatment have been reported since 2000,^9^ revealing an overall vulnerability of this population stemming from generational patriarchal values and age-related illnesses.^10^ Characteristics of sexual mistreatment resemble those of intimate partner or domestic mistreatment, with survivors perceived as easy to manipulate. Older adults’ sexual mistreatment risk characteristics include:^11^

• The survivor’s age is later adult life
• Lower generation power
• Cognitive Impairment
• The survivor is older than the perpetrator
• The role of sexual activity is viewed as asexual
• Very little public awareness and interest
• Few services available for assistance
• Legislative responses: Mandatory reporting and penalty enhancements
• Little in academic research
• Survivor’s reluctance to report because of shame & and embarrassment

Perpetrators come from various socioeconomic backgrounds, targeting the vulnerability of older adults and using their domineering behaviors to derive pleasure through humiliation and inflicting pain.^11^ Some data suggested that perpetrators of older adult sexual mistreatment have a history of prior criminal offenses and maybe younger males aged 16 to 31.^12^ Sexual-related crimes have also been reported to be more violent and occur more often in older adult residences.^13^ Although sexual-related crimes are commonly more violent than other crimes, older adult sexual mistreatment more often occurs in their *home* by someone the older adult knows, lowering the likelihood of detection by authorities and increasing the possibility of shame, embarrassment, and concealment by the older adult.

Sexual violence in older adults includes harassment, demands for sex in exchange for food or medication, assistance with hygiene or caregiver responsibilities, nonconsensual exposure to pornography, genitalia, and genital mutilation, exacerbating medical and psychological problems, leading to legal disputes.^2^ The cultural stigma linking sexual desirability to attractiveness may contribute to overlooking older adults as targets for sexual violence. Still, research emphasizes that sexual violence is rooted in oppression rather than sexual attraction.^4^ Overall, addressing sexual mistreatment as a multifaceted issue requires a comprehensive approach that considers the complexities of medical, psychological, and legal factors.

## Strategies and evidence

A recent study estimated that 14% of noninstitutionalized older adults had suffered physical, psychological, or sexual mistreatment. Furthermore, women with disabilities are 4 times more likely to experience sexual assault than women without disabilities.^14^ The broader implications of older adult mistreatment include hospitalization, mental health, coroners, premature deaths, and the development of protocols and programs to address its effects that have resulted in substantial costs of billions of US dollars for society. Older adult mistreatment, including sexual abuse, accounts for 0.8% of the expenses attributed to hospitalizations, medical and mental health expenses, medical examiner costs, court expenses, guardianship expenses, and other associated costs aimed at protecting and providing for survivors.^2,5^

Addressing the sexual mistreatment of older adults requires sensitivity and an evidence-based approach. Trauma-informed care principles are preferred and critical in getting older adults to agree to self-report mistreatment during screening and to cooperate with proposed interventions. The principles center around “(1) building rapport and approaching the older adult with compassion and care, (2) setting the context before asking the [older adult] questions that may arouse shame and embarrassment, and (3) allowing mutuality, collaborative work, and shared decision-making during the response.”^15^ Implementing these practices is essential with every individual encounter with the older adult sexual mistreatment survivor.

This approach should be taught as a best practice to enhance identification and promote long-term care and safety for older adults. Additionally, more older adults will likely reveal information by applying these practices. Better data regarding the risks, indicators, and consequences will emerge, improving future older adult sexual mistreatment identification and response and intervention and prevention efforts. Targeting these goals increases the potential to create a more effective system for managing and preventing sexual mistreatment in the older adult population.

As with Mary, given the characteristics that increase the risk for sexual mistreatment, the age of the woman, perceived position of less power, physical disabilities, and cognitive impairment lead to conjecture of her as asexual. In this case, mandatory reporting and penalty developments can benefit survivors and others from victimization.

Increasing national awareness is an essential step to decreasing the problem, as has been seen historically with child abuse and domestic violence. Cognizance will similarly advocate for better approaches to help diminish older adult mistreatment. Additionally, legal advocacy is critical for decreasing older adult mistreatment, as litigations against the perpetrators will hold them accountable and impact society systemically.^11^

Education regarding the recognition and reporting of older adult sexual mistreatment is crucial for primary care physicians, psychiatrists, social workers, and other health care professionals. Despite growing national awareness and interest in elder mistreatment, there remain limited resources published to guide elder sexual mistreatment educational interventions.^11^ These are sorely needed and could benefit many first responders and health care professionals, including psychiatrists. For example, short online modules about identification, interviewing, and responding to older adult sexual mistreatment could be developed like the those that exist for different forms of child abuse and now for identifying and responding to elder mistreatment in emergency departments through applications such as the elder mistreatment emergency department toolkit (https://gedcollaborative.com/toolkit/elder-mistreatment-emergency-department-toolkit/).

## Diagnosis and evaluation

Psychiatrists and clinicians may be unfamiliar with the assessment for sexual mistreatment of older adults. Practitioners focusing on sexual assault are known as Sexual Assault Forensic Examiners (SAFEs), encompassing Registered Nurses who specialize in the Forensic Nursing subspecialty and serve in the role of Sexual Assault Nurse Examiners (SANEs).

Typically, clinicians may find conditions that mimic conditions of aging that are pretty typical for sexual mistreatment, such as those associated with chronic illness. These conditions comprise:^6^

• Cystoceles or uterine prolapse
• Decreased anal sphincter function
• Fixed drug eruption
• Inflammatory bowel disease
• Perineal excoriation from incontinence or lichen sclerosis
• Vaginal bleeding and excoriation from low estrogen
• Vaginitis

It is advisable to assess the survivor independently from their caregiver, given that caregivers or relatives are frequently identified as the most common perpetrators. Survivors may exhibit signs of fear towards the caregiver, express refusal or reluctance to be alone with them, or may be found lingering around populated areas like the nurse’s station. Psychological indicators of sexual mistreatment encompass behavioral changes that may also include agitation, alterations in social interactions, withdrawal, depression, signs of post-traumatic stress disorder, the development of anxiety or panic attacks, suicide attempts, or the emergence of new or atypical sexual behaviors.^16^

Suspicious indicators of sexual mistreatment during physical examination include^5,11,17^

• Difficulty sitting or walking
• Ecchymosis around the genital area and/or breasts
• Underwear is torn or stained
• Unexplained trauma: vaginal, anal, or rectal
• Unexplained sexually transmitted infections or genital infections
• Verbal report or Observation of sexual mistreatment
• Change in toileting behavior
• Unusual marks on ankles, wrists, and neck
• Imprints of hands, fingers, belts, ligature or bite marks

The increase in fragility and decrease in lubrication of the vulva and vaginal areas in older adult women increase the risk of genital injury in sexual assault.^17^ The reduction in the vaginal tissue plays a role secondary to the reduction of hormone production from menopause and also increases the risk of injury with consensual or nonconsensual intercourse.^18^

Discussing sexual mistreatment requires a sensitive and empathic approach. It is crucial to build a rapport through an introduction, seeking permission, and ensuring the person’s well-being and safety. Beginning the conversation with open-ended and indirect questions, like asking about the home environment, can help the individual feel more at ease, for example, “Can you share what your home environment is like?” Then, the interviewer can move on to more direct open-ended questions such as, “Has anyone made offensive comments to you or touched you sexually without your consent?” (Refer to Table 1).^19^

**Table 1.** Specific questions for asking about sexual mistreatment.^19^

• Have you been forced to have sex even if you did not want to?
• Have you been forced to have sex in a way that you do not want to?
• Has anyone touched your genitals without your consent?
• Have you been raped?
• Has anyone taken pictures of you naked without your consent?
• Have you been forced to watch sexual programs or videos that make you feel uncomfortable?

Sexual mistreatment constitutes a clinical diagnosis relying on history and physical examination. Accidental bruising is common in older adults, and it is essential to distinguish this from symptoms that overlap with normal aging, such as senile ecchymosis. Approximately ninety percent of unintentional bruises are found on the extremities. This pattern contrasts with the bruises associated with sexual mistreatment, which are commonly found in the neck, ears, genitalia, buttocks, or soles of feet.^20^

## Management

The primary focus in managing older adults suspected of sexual mistreatment is ensuring patient safety. Clinicians recommend utilizing local resources, such as shelters, home care agencies, legal and police services, and government-supported older adult mistreatment services, ie, Adult Protective Services (APS). Interdisciplinary care by physicians, psychiatrists, nurses, and other clinicians, and occasionally, family members, plays a crucial role in assisting survivors of sexual mistreatment. Management of sexual assault includes its differentiation, as sexual assault is likely unrecognizable and often overlooked. Mandatory reporting to APS is required, with some interstate variation. Measurement of occurrence, recurrence, and interventions has been reported as complicated in the maltreatment of older people, and it is unclear whether improvement in numbers is secondary to better reporting or an increase in recurrence.^21^

Comprehensive strategies should address the legal issues and the survivor’s health, capacity, physical condition, and social support. Investigational challenges may arise due to cultural, language, and religious differences. Recognizing the importance of person-centered care includes identifying older adults as individuals. It is vital to avoid ageism and stereotypes that may exclude them from conversations and decision-making.^11^ Building trust through acknowledgment of negative feelings such as shame, helplessness, fear, and vulnerability^22^ may be bridged by incorporating more specific questions for asking about sexual mistreatment (see Table 1). The situation’s complexity may lead the survivor to maintain a connection with the perpetrator or alter their story for self-protection. Most importantly, trauma-informed principles, like ensuring privacy and comfort^15^ and providing assistance and reassurance to the survivor, are paramount.^11^

In sexual mistreatment cases involving a nursing facility, an ombudsperson plays a crucial role in the investigation to safeguard each facility resident. APS coordinates professionals’ roles in law enforcement, Medicaid Fraud unit, forensic nursing, local and state regulatory agencies, and survivors’ advocates, ensuring the protection and privacy of the survivor.^11^

SAFEs and SANEs provide survivor-centered, trauma-informed care to individuals who have experienced violence and abuse. Collaborating with advocates, law enforcement, attorneys, and other health care providers ensures the patient’s needs are comprehensively addressed. When an older adult survivor of violence presents to an emergency department or a sexual assault center, the SAFE or SANE conducts the medical-forensic examination.^23^

The medical-forensic examination includes a thorough examination that covers treatment and evidence collection needs, including triage, medical screening, history, physical assessment, evidence collection, and discharge planning.^24^ The SAFE/SANE gathers information, history, and details from the patient about the circumstances of the assault, documenting details through narrative descriptions, diagrams, and photography to capture physical injuries. In cases of sexual assault, evidence is collected using the sexual assault evidence collection kit (SAK). Prophylactic treatment is administered to minimize the risk of exposure to sexually transmitted infections. Psychological considerations for the patient include addressing safety, collaborative decision-making, and empowerment.^24^

The SAFE or SANE are obligated to make mandatory reports to protective services agencies as per state requirements and may testify as experts in courts of law regarding the medical-forensic examination, evidence collected, and any injuries the patient sustained during the assault.^16^

In addition to the physical trauma, many survivors suffer psychological distress. They may be diagnosed with psychiatric conditions such as post-traumatic stress disorder or depression. Providers may choose their words wisely (refer to Table 2) when communicating with survivors of sexual mistreatment, providing language that supports individuals to deal with their emotions.^17,25-25^

**Table 2.** Communication and language to support survivors of sexual violence in dealing with their emotions.^17,25,26^

	Signs and Symptoms	Recommendation
Immediate response		
Shock and numbness	Feelings of disbelief or denial Crying uncontrollably, laughing nervously, withdrawing or claiming to feel “fine” or nothing	Recommend talking with a friend, praying if religious, and having people around that make the person feel safe Try to normalize by saying, “this is a common reaction to severe trauma.”
Response in weeks to months		
Disruption of daily life	Difficult concentrating or difficulty not thinking about the event Nightmares, trouble sleeping, appetite changes, anxiety, and/or depression	Recommend to sleep in another room if victimized trauma occurred in that room. Make small changes, such as taking a different route to a common destination Recommend stress management techniques
Loss of control	Racing and/or overwhelming thoughts A person may feel anxious, jittery, and scared Difficulty with focus or concentration	Recommend making small decisions on their own Recommend talking to a trusted friend Seek out professionals such as counselors or legal advice
Fear	Fear of the perpetrator Fear of being alone Fear of people or situations that remind the person of the event Fear can come and go and range from mild to severe anxiety	Recommend changes that will make a person feel safe. For example, change locks, take a self-defense class, or stay with a trusted friend or family. If fear interferes with life, the recommendation is to seek a professional counselor
Guilt and shame	Recurrent thoughts that the person could have done something different to avoid or prevent the event Self-doubt of judgement or trust of instincts	Remind the survivor that they could not have caused this event to happen. Recommend reading about recovering from rape “You are not to blame for what happened to you.”
Anger	Anger can be directed towards anyone, including the perpetrator, policy, family, medical staff, counselor, church/religion, and self	Recommend that a person allow anger, as it is a natural and common reaction. Recommend physical activity such as exercise to release tension. Recommend to play music or sing. Recommend support groups. If religious, recommend contacting a spiritual leader such as a rabbi, minister, or priest
Isolation	Avoidance of spending time with people. Withdrawal or distancing from friends and family	Reassurance that the victim survivor is not alone in what they are feeling. Recommend support groups or counseling. Recommend reading for validation of feeling
Vulnerability and mistrust	Feelings of trust and safety are absent. Feelings of being guarded, suspicious, and/or cautious around others. Feelings of questions own judgment	Recommend turning to people, they depend on, such as friends and family. Recommend people who are good listeners and take time in relationships Ask, “can you think of some trustworthy people in your life right now?”

## Prevention

The USPSTF has not identified supporting evidence indicating that screening and early detection of older adult mistreatment reduces occurrence. The Grade I* USPSTF recommendation states that there is insufficient evidence to determine the risks and benefits of screening all older or vulnerable adults for mistreatment. Despite the lack of clear evidence for universal screening, health care professionals have professional and legal obligations to appropriately recognize, diagnose, report, and refer to the mistreatment of older adults.^6^

**Figure 1.**
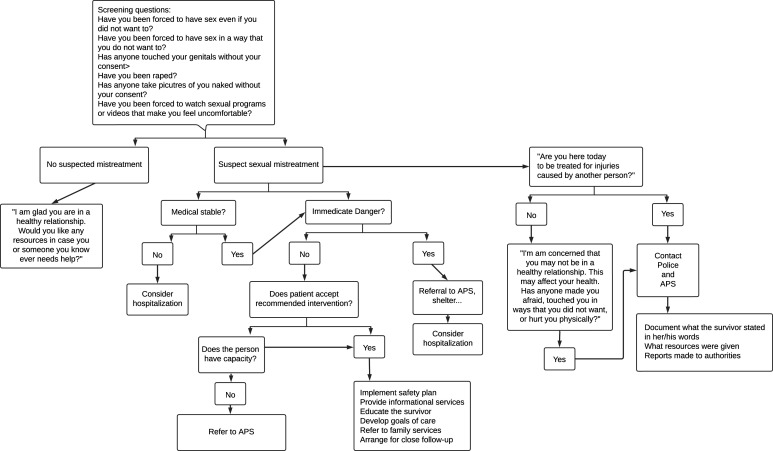
Algorithm for management of sexual mistreatment.^6,27^

Managing sexual mistreatment poses challenges due to its understudied and underreported nature. Identifying characteristics and risk factors associated with the perpetrator can aid in linking the survivor with the event. Yet, the consequences and management of the subsequent steps might not be straightforward. Medical providers are tasked to protect the patient, and while state statutes may vary, mandatory reporting is legally binding. Even in nursing homes, allegations of mistreatment must be investigated and reported. An algorithm (refer to Figure 1^6,26^) will help you comply with mandatory reporting requirements.

## Areas of uncertainty

Numerous studies highlight the many challenges in researching older adult mistreatment, revealing considerable gaps in this field. Ethical dilemmas, Institutional Review Boards (IRBs), standardized evidence-based study designs, and shortage of researchers contribute to the difficulty in obtaining robust and concrete data. Unfortunately, there remains a lack of substantial data on sexual mistreatment in older adults, and overall research on older adult mistreatment is limited. Gill (2021) suggests that individuals faced with mistreatment have barriers to seeking help, such as lack of awareness, self-blame, shame and fear, and dependency level. Eighty-three percent of older adult survivors reported dependency on their perpetrators due to financial stress or underlying medical conditions such as physical or cognitive impairments.^27^

Further evaluation of the accuracy and acceptability of screening and disclosure is essential. High-quality randomized and controlled trials focusing on screening and interventions are vital to improve reporting and patient safety, strengthen support systems, and address legal implications. Studies continue to emphasize the gaps in older adult mistreatment research. The emphasis is to move the field of older adult sexual mistreatment forward by raising awareness and changing attitudes, providing health providers tools to improve reporting, reducing the barriers to seeking help, and educational interventions such as approaches like person-centered and trauma-informed care.

## Conclusions and recommendations

The vignette illustrates the profound impact of sexual mistreatment on the woman, Mary, emphasizing the need for proactive measures to protect individuals like her. Vigilant staff selection and training, adequate staffing, better supervision of patients with violent tendencies, and controlled visitor policies contribute to patient safety. Implementation of clinician suspicion and policies encouraging incident reporting, along with responsiveness and availability of management, is crucial.

Long-term consequences of sexual mistreatment extend beyond the acute incident, affecting individuals physically, emotionally, and financially. Addressing this loss of independence is vital, as it impacts healthcare costs for the patient and the nation. More research can be explored in older adult mistreatment prevention. The loss of autonomy contributes to the costs for the Medicare system, involving about 34 billion hours of unpaid care for older adults, estimated at $470 billion annually.^28^ For example, the International Network for Prevention of Elder Abuse (INPEA) outlines efforts to promote education and training of health care professionals, advocacy, and research. The INPEA instituted World Elder Abuse Awareness Day in 2006, and by 2011, the United Nations recognized older adults’ mistreatment as a global issue impacting millions of older people. Efforts from the United States Government included the Elder Justice Act and Policy-Level and endorsement of $777 million to Adult Protect Services to improve data collection, training programs, ombudsman programs, and grants. Workshops to prioritize public health response suggested seven actions:^29^

• Create a policy to recognize elder abuse as a public health issue
• Challenge current research priorities vital in updating policy and practice, such as interventions, achievements, prevention, data collection, and cost
• Transform what we know into practice
• Focus on the resources issue
• Impose the law and build policy infrastructure
• Construct a governmental constituency, and
• Sponsor and support advancement and innovation.

Despite research endeavors and public policy, medical professionals must utilize and build stronger relationships with APS. Psychiatrists and medical professionals must recognize that sexual mistreatment occurs in later life and is under-reported and create a safe space for protecting patients in these highly sensitive situations. Given the mandatory reporting role, collaborations with APS and law enforcement become crucial to facilitate a person-centered, multidisciplinary approach for older adults to ensure safety beyond the person discussed in this case.
